# A Prediction Model for Tumor Recurrence in Stage II–III Colorectal Cancer Patients: From a Machine Learning Model to Genomic Profiling

**DOI:** 10.3390/biomedicines10020340

**Published:** 2022-02-01

**Authors:** Po-Chuan Chen, Yu-Min Yeh, Bo-Wen Lin, Ren-Hao Chan, Pei-Fang Su, Yi-Chia Liu, Chung-Ta Lee, Shang-Hung Chen, Peng-Chan Lin

**Affiliations:** 1 Department of Surgery, National Cheng Kung University Hospital, College of Medicine, National Cheng Kung University, Tainan 704, Taiwan; n040581@mail.hosp.ncku.edu.tw (P.-C.C.); linbw@mail.ncku.edu.tw (B.-W.L.); n803421@mail.hosp.ncku.edu.tw (R.-H.C.); 2 Department of Oncology, National Cheng Kung University Hospital, College of Medicine, National Cheng Kung University, Tainan 704, Taiwan; s98031083@mail.ncku.edu.tw; 3 Department of Statistics, National Cheng Kung University, Tainan 704, Taiwan; pfsu@ncku.edu.tw (P.-F.S.); z10907004@ncku.edu.tw (Y.-C.L.); 4 The Center for Quantitative Sciences, Clinical Medicine Research Center, National Cheng Kung University Hospital, Tainan 704, Taiwan; 5 Department of Pathology, National Cheng Kung University Hospital, College of Medicine, National Cheng Kung University, Tainan 704, Taiwan; lcta@mail.ncku.edu.tw; 6 National Institute of Cancer Research, National Health Research Institute, Tainan 704, Taiwan; 7 Department of Genomic Medicine, National Cheng Kung University Hospital, College of Medicine, National Cheng Kung University, Tainan 704, Taiwan; 8 Department of Computer Science and Information Engineering, College of Electrical Engineering and Computer Science, National Cheng Kung University, Tainan 704, Taiwan

**Keywords:** machine learning model, colorectal cancer, cancer recurrence, age, lymph node ratio

## Abstract

Background: Colorectal cancer (CRC) is one of the most prevalent malignant diseases worldwide. Risk prediction for tumor recurrence is important for making effective treatment decisions and for the survival outcomes of patients with CRC after surgery. Herein, we aimed to explore a prediction algorithm and the risk factors for postoperative tumor recurrence using a machine learning (ML) approach with standardized pathology reports for patients with stage II and III CRC. Methods: Pertinent clinicopathological features were compiled from medical records and standardized pathology reports of patients with stage II and III CRC. Four ML models based on logistic regression (LR), random forest (RF), classification and regression decision trees (CARTs), and support vector machine (SVM) were applied for the development of the prediction algorithm. The area under the curve (AUC) of the ML models was determined in order to compare the prediction accuracy. Genomic studies were performed using a panel-targeted next-generation sequencing approach. Results: A total of 1073 patients who received curative intent surgery at the National Cheng Kung University Hospital between January 2004 and January 2019 were included. Based on conventional statistical methods, chemotherapy (*p* = 0.003), endophytic tumor configuration (*p* = 0.008), TNM stage III disease (*p* < 0.001), pT4 (*p* < 0.001), pN2 (*p* < 0.001), increased numbers of lymph node metastases (*p* < 0.001), higher lymph node ratios (LNR) (*p* < 0.001), lymphovascular invasion (*p* < 0.001), perineural invasion (*p* < 0.001), tumor budding (*p* = 0.004), and neoadjuvant chemoradiotherapy (*p* = 0.025) were found to be correlated with the tumor recurrence of patients with stage II–III CRC. While comparing the performance of different ML models for predicting cancer recurrence, the AUCs for LR, RF, CART, and SVM were found to be 0.678, 0.639, 0.593, and 0.581, respectively. The LR model had a better accuracy value of 0.87 and a specificity value of 1 in the testing set. Two prognostic factors, age and LNR, were selected by multivariable analysis and the four ML models. In terms of age, older patients received fewer cycles of chemotherapy and radiotherapy (*p* < 0.001). Right-sided colon tumors (*p* = 0.002), larger tumor sizes (*p* = 0.008) and tumor volumes (*p* = 0.049), TNM stage II disease (*p* < 0.001), and advanced pT3–4 stage diseases (*p* = 0.04) were found to be correlated with the older age of patients. However, pN2 diseases (*p* = 0.005), lymph node metastasis number (*p* = 0.001), LNR (*p* = 0.004), perineural invasion (*p* = 0.018), and overall survival rate (*p* < 0.001) were found to be decreased in older patients. Furthermore, *PIK3CA* and *DNMT3A* mutations (*p* = 0.032 and 0.039, respectively) were more frequently found in older patients with stage II–III CRC compared to their younger counterparts. Conclusions: This study demonstrated that ML models have a comparable predictive power for determining cancer recurrence in patients with stage II–III CRC after surgery. Advanced age and high LNR were significant risk factors for cancer recurrence, as determined by ML algorithms and multivariable analyses. Distinctive genomic profiles may contribute to discrete clinical behaviors and survival outcomes between patients of different age groups. Studies incorporating complete molecular and genomic profiles in cancer prediction models are beneficial for patients with stage II–III CRC.

## 1. Introduction

Colorectal cancer (CRC) is the third-most prevalent type of cancer worldwide, with approximately two million newly diagnosed cases reported in 2020 [1]. In Taiwan, CRC is the second most common cancer type, with a crude incidence rate of 70.05 per 100,000 people found in 2018 [2]. Moreover, CRC is the second-leading cause of cancer-related deaths globally and accounted for approximately 900,000 deaths in 2020 [1]. Although surgery can be chosen as a curative procedure, disease recurrence can affect the survival of patients with CRC. Several reports show that the five-year postoperative recurrence rates for patients with stage II and III CRC are around 10–15% and 25–30%, respectively [3,4,5,6,7,8]. Therefore, it is crucial to identify the predictors of cancer recurrence for patients with stage II–III CRC after surgical resection for appropriate adjuvant therapies, especially for patients with a high risk of recurrence. Several histopathological features have been reported to be correlated with tumor recurrence in patients with stage II–III CRC [5,6,7,8]. According to the National Comprehensive Cancer Network guidelines, pathological stage T4, poorly differentiated histology, perineural invasion (PNI), lymphovascular invasion (LVI), inadequately harvested lymph nodes (<12 lymph nodes), and positive surgical margins are classified as high-risk factors for tumor recurrence in these patients [5]. Due to the increasing complexity of modern cancer treatments, the pathology reports of resected CRC specimens encompass basic clinicopathological characteristics and pivotal histopathological features for prognostication. In addition to the standardized diagnosis of CRC, modern pathology reports can facilitate personalized treatment and the exchange of information in multicenter clinical trials or international studies [9,10].

During the past decade, the construction of a risk prediction model for CRC recurrence has been a popular and appealing task. By combining histopathological features from pathology reports with clinical characteristics, some actionable nomograms have been reported for the prediction of CRC recurrence [8,11]. Due to their distinct research designs and driving hypotheses, these prediction models, based on conventional statistical methods, have limited external validity [12]. Therefore, machine learning (ML) applications have emerged as approaches to integrate multiple risk factors into a predictive algorithm for patients with cancer [13]. ML algorithms are designed to systematically handle large sample sizes with complex features, intricate interactions, and non-linear properties. In contrast to conventional statistical models, ML processes can automatically model the data generating process and estimate feature coefficients with fewer assumptions. Currently, several ML algorithms, including classification and regression trees (CARTs) and support vector machines (SVMs), are widely applied for the analysis of the histopathological data of CRC specimens [14,15]. In addition, some studies have demonstrated the feasibility of integrating statistical and ML algorithms to obtain a higher predictive performance in determining cancer prognosis [15,16]. Hence, the development of ML models is a promising application for the prediction of CRC recurrence and potential personalized treatment.

To date, several studies have generated promising results by using ML strategies to analyze various clinicopathological features to predict cancer recurrence and the survival of patients with CRC [12,13,14,15,16]. However, the CRC datasets examined in these predictive studies are usually across all disease stages, and studies focusing on stage II and III CRC are limited. In this study, we aimed to investigate tumor recurrence using established ML algorithms based on histopathological features and clinical characteristics from standardized pathology reports and medical records from resected stage II–III CRC specimens at National Cheng Kung University Hospital (NCKUH). We quantized the performance of several ML models and built a predictive nomogram for clinical application. During these processes, we identified older age as a significant predictor of tumor recurrence. To further evaluate the molecular mechanisms underlining the aging process that was correlated with tumor recurrence, we evaluated the genomic profiles of CRC specimens.

## 2. Material and Methods

### 2.1. Study Population and Histopathology

This cohort study included patients with pathological stage II–III CRC who received curative surgical resection at NCKUH between January 2004 and January 2019. All enrolled patients signed informed consent statements prior to the surgery and received standard surgical resection with a complete pathology report. Clinical information and histopathological features were identified from electronic medical records and standard pathology reports, respectively. Following NCCN guidelines, the decision to initiate neoadjuvant and adjuvant chemotherapy depends on clinical and pathological stages and the shared decision-making discussions between patients and physicians. Stage III patients typically received oxaliplatin-based mFOLFOX6 (5-fluorouracil, leucovorin, and oxaliplatin) adjuvant chemotherapy, and high-risk stage II patients received 5-FU-based adjuvant chemotherapy. 

For the assessment of histopathological features, tumor configuration was decided macroscopically. “Exophytic” indicated that the tumor was polypoid grossly; “endophytic” indicated that the tumor was ulcerative. The criteria of histologic grade were defined as follows: well-differentiated (>95% gland formation), moderately differentiated (50–95% gland formation), and poorly differentiated (<50% gland formation). Tumor status was recorded according to the TNM staging system. pT status was determined by the tumor invasion levels; pN status was determined by the numbers of regional lymph nodes with tumor metastasis. Lymphovascular invasion was considered when the tumor cells invaded the lymphovascular channels microscopically; the perineural invasion was considered when the tumor cells invaded the nerve bundles microscopically. The “infiltrating” tumor growth pattern was defined as tumor cells with streaming dissection of muscularis propria or mesenteric adipose tissue microscopically, otherwise the tumor was “pushing”. “Tumor budding” was reported when individual tumor cells or small clusters (<5 cells) of tumor cells separated from the main tumor were identified at the invasive front. Tumor regression grade (TRG) was used for evaluation of the treatment effect of neoadjuvant therapy. The criteria were defined as follows: TRG1 (no residual tumor), TRG2 (rare residual tumor cells), TRG3 (fibrosis outgrowing residual tumor), TRG4 (residual tumor outgrowing fibrosis), and TRG5 (absence of regressive change). Immunohistochemistry (IHC) was performed on formalin-fixed paraffin-embedded tissues using a standard avidin-biotin complex peroxidase procedure with an autostainer (Benchmark XT, Ventana, Tucson, AZ, USA). In brief, four µm-thick tissue sections were obtained from a representative formalin-fixed, paraffin-embedded tissue block of each tumor. The sections were deparaffinized and rehydrated, and heat-induced epitope retrieval was performed using the cell conditioning solution CC1 (Ventana, Tucson, AZ, USA). The slides were incubated with primary antibodies for MLH1 (clone: M1; diluted 1:1; Ventana), PMS2 (clone: EPR3947; diluted 1:1; Ventana), MSH2 (clone: G219-1129; diluted 1:1; Ventana), MSH6 (clone: 44; diluted 1:1; Ventana), or BRAF V600E (clone: VE1; diluted 1:1; Ventana). These proteins were detected and visualized using a Roche OptiView DAB IHC Detection Kit (Ventana). The slides were counterstained with hematoxylin and coverslipped. Positive and negative controls were included in all of the runs. Negative controls omitted the primary antibodies, and positive controls were tissues known to express the proteins. All the IHC stain CRC samples were reviewed by a pathologist (Lee, C.T.). The expression of four MMR proteins (MLH1, PMS2, MSH2, and MSH6) was defined as abnormal (loss) when the nuclear staining of tumor cells was absent despite positive staining being seen in the surrounding stromal cells. dMMR (deficient MMR) was defined as the loss of at least one MMR protein in tumor cells. pMMR (proficient MMR) was defined as the expression of all four MMR proteins in tumor cells. This study was approved by the Institutional Review Board of NCKUH (B-ER-110-342). 

### 2.2. Data Collection

CRC-specific standardized pathology reports, which involved the assessment of resected CRC specimens and were recorded by CRC-specialized pathologists at NCKUH, were collected for variable standardization. The variables in the pathology report included tumor location, surgical procedure, neoadjuvant chemoradiotherapy (CCRT), macroscopic tumor configuration, tumor size, histology type and grade, TNM stage, adequacy of excision (i.e., distal and circumferential margins), LVI, PNI, tumor deposits, growth pattern at tumor periphery, tumor budding, and additional pathological findings. Clinical features, including age, sex, body mass index (BMI), and additional treatments, were retrieved from medical records.

### 2.3. ML Models and Nomogram

Four ML models based on logistic regression (LR), random forest (RF), CART, and SVM were used for predicting tumor recurrence. For ML processing, K-fold cross validation was performed (K = 5); the data were randomly split into 80% and 20% for the training and independent testing models, respectively. The caret R package (R version 4.0.5; R studio version 1.4.1106) [17,18] was employed to build all the ML models, including the “stat” R package for LR, the “randomForest” R package for the RF, the “rpart” R package for CART, and the “e1071” R package for SVM. The links for the R packages were as follows: Randomforest: https://cran.r-project.org/web/packages/randomForest/randomForest.pdf (accessed on 2 December 2021); CART: https://cran.r-project.org/web/packages/rpart/rpart.pdf (accessed on 2 December 2021); SVM: https://cran.r-project.org/web/packages/e1071/e1071.pdf (accessed on 2 December 2021). Variables with missing values greater than 5% of the dataset were excluded from these analyses. If the remaining variables had missing values, the multivariate imputation by chained equations method was applied to fill in the dataset. We used fraction of missing information (FMI), which represents the proportion of the total sampling variance that is due to missing data, to evaluate the risk of imputation. The FMI of variables used in logistic regression was in the range of 0.002 to 0.009, which was very small. In other words, this means that only 0.2~0.9% of the total sampling variance was attributable to missing data. To estimate each model’s performance, the area under the curve (AUC) of the receiving operating characteristics, accuracy, sensitivity, specificity, and F1 score was derived from five-fold cross-validation with 100 repeats.

A nomogram representing a graphical calculation instrument based on the Cox logistical regression model was constructed for clinical prognostication [19]. The effects of prognostic factors on cancer recurrence were defined in the format of axes, and risk points were attributed according to the prognostic variables. Total scores for each patient were calculated by adding the individual scores of all five risk factors based on the nomogram. In this nomogram analysis, the probability of recurrence increased as the total score increased. For a CRC patient who had a tumor sized ≥6 cm, the corresponding risk was approximately 30 points. If the lymph node ratio (LNR) was 0.5, the corresponding risk was approximately 53 points. If PNI was unidentified (absence), the corresponding risk was 0 points. If patients received neoadjuvant CCRT, the corresponding risk was approximately 55 points, and if patients were older than 65 years the corresponding risk was approximately 25 points. Using this nomogram system, when the “Total Points” amounted to 163 the probability of a patient’s cancer recurrence was 0.57 based on the LR model.

### 2.4. Tumor Sequencing with a Targeted Gene Panel

A total of 123 patients with primary histologically confirmed stage II–III CRC tumor samples obtained from standard surgical resection from National Cheng Kung University Hospital (NCKUH) were recruited for this study between January 2014 and January 2019 and were subjected to a histopathological assessment followed by nucleic acid extraction from formalin-fixed, paraffin-embedded blocks at NCKUH. All participants signed written informed consent statements, and clinical information was obtained from electronic medical records. Independent pathologists reviewed the specimens, examined the percentage of viable tumor nuclei, and determined the feasibility of the mutational profile detection for each specimen. The deep targeted sequencing of tumor samples was performed using Oncomine Comprehensive Assays (Thermo Fisher Scientific, Waltham, MA, USA). This test was designed to evaluate somatic variants within 115 druggable genes. Data quality control, alignment, variant calling, and limit of detection (LOD) calculation were conducted using a locked data analysis pipeline, with the workflow in the Torrent Suite Software 5.0.4 (Thermo Fisher Scientific, Waltham, MA, USA). All reads were aligned to the hg19 reference genome, and variant calling was performed using the Torrent Variant Caller plugin (version 5.0.4.0; Thermo Fisher Scientific, Waltham, MA, USA). Variant annotation was performed using annovar version: 8 June 2020 [20]. To identify potential cancer driver mutations, we used a populational allele frequency cutoff of 1% with respect to the Taiwan Biobank, gnom AD, and 1000 G databases [21]. We selected exonic single-nucleotide variants (SNVs) and splicing genetic variants for analysis.

### 2.5. Statistical Analysis

The data analysis was performed by the Center for Quantitative Sciences. We tabulated the values of descriptive statistics, including means and standard deviations for continuous variables and percentages and frequencies for categorical variables. To analyze the baseline characteristics of patients with CRC across age, the Kruskal–Wallis and Wilcoxon rank-sum tests were used for continuous variables. By contrast, Fisher’s exact test was used for categorical variables (e.g., sex, tumor location, etc.). Moreover, the Kaplan–Meier method and log-rank tests were used for time-to-event endpoints. Recurrence-free survival (RFS) was the duration between the date of the surgical resection of CRC and the date of detecting any cancer recurrence at either local, regional, or distant locations. Overall survival (OS) was the time from the date of diagnosis until death by any cause. Cox proportional hazards models that included all baseline variables were employed for the analysis of RFS and OS. The odds ratios, hazard ratios, and 95% confidence intervals were estimated with this model. All statistical tests were two-sided, and a *p*-value of < 0.05 indicated statistical significance. All analyses were performed using the R statistical software (version 4.0.2) for Windows.

## 3. Results

### 3.1. Clinical Characteristics and Histopathological Features of Patients with and without Cancer Recurrence

A total of 1073 CRC patients were enrolled in this study. The baseline characteristics and histopathological features of all patients are summarized in Table 1. Among all patients, 159 (14.8%) developed cancer recurrence, including 13 who developed local recurrence (8.2%) and 146 (91.8%) who developed distal recurrence. Recurrence occurred more frequently in men (*n* = 102, 64.2%) than in women (*n* = 57, 35.8%), although this result was not significant (*p* = 0.062). Among all clinicopathological features, chemotherapy (*p* = 0.003), endophytic tumor configuration (*p* = 0.008), TNM stage III disease (*p* < 0.001), pT4 (*p* < 0.001), pN2 (*p* < 0.001), increased LNR (*p* < 0.001), LVI (*p* < 0.001), PNI (*p* < 0.001), tumor budding (*p* = 0.004) and neoadjuvant CCRT (*p* = 0.025) were correlated with higher rates of tumor recurrence. The survival rate of patients with tumor recurrence was lower than that of patients without recurrence (*p* < 0.001). 

### 3.2. ML Model Performance for Assessing CRC Recurrence and Predictive Nomogram

**Figure 1:**
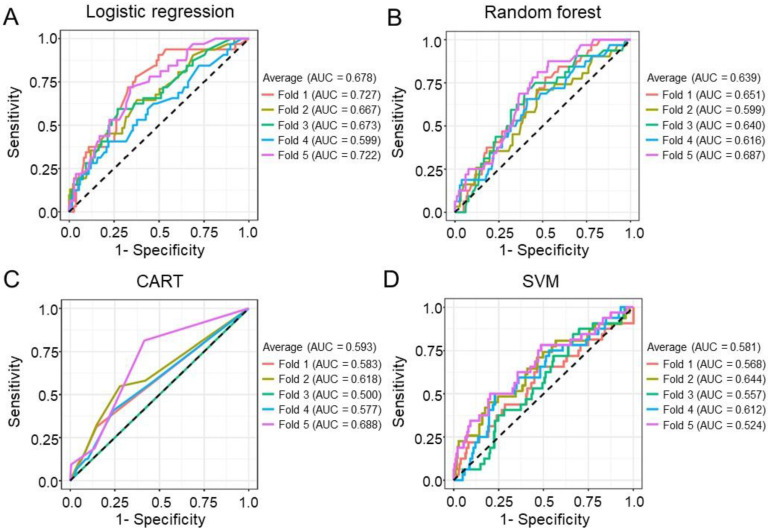
Prediction model with stratified five-fold cross-validation for cancer recurrence. (**A**) A logistic regression model was used to predict cancer recurrence with the highest average area under the curve (AUC) = 0.678. (**B**) A random forest model was used to predict cancer recurrence with an average AUC = 0.639. (**C**) Classification and regression decision trees (CARTs) were used to predict cancer recurrence with an average AUC = 0.593. (**D**) A support vector machine (SVM) was used to predict cancer recurrence with an average AUC = 0.581.

**Figure 2:**
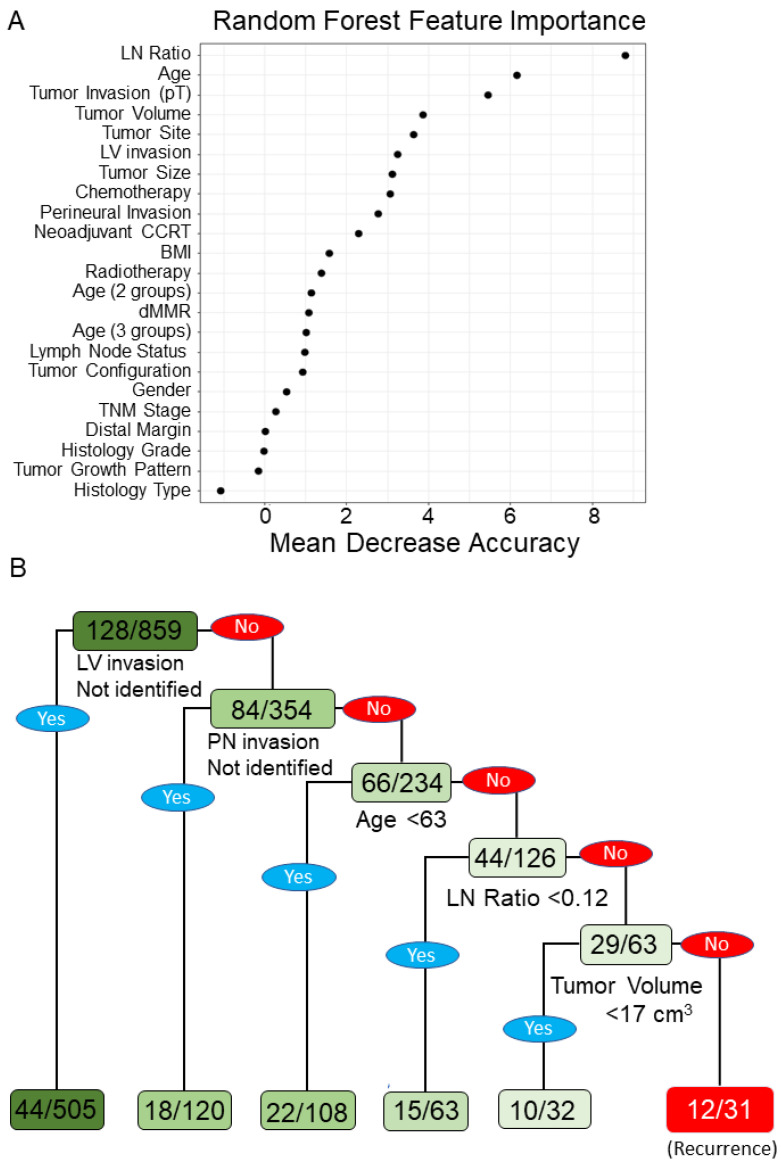
Prognostic factors in the random forest (RF) model and CART decision tree analysis. (**A**) The importance of factors in terms of the mean decrease in accuracy in the RF model. We ranked the relative importance for all these prognostic factors. From the results, the top five factors that corresponded to cancer recurrence were the lymph node ratio, age, depth of tumor invasion stage (pT), tumor volume, and tumor site. (**B**) A decision tree was generated by the CART model to optimize the risk stratification in cancer recurrence. The internal nodes of the tree represent prognostic factors, including lymphovascular (LV) invasion, perineual (PN) invasion, age, lymph node ratio, and tumor volume.

**Figure 3:**
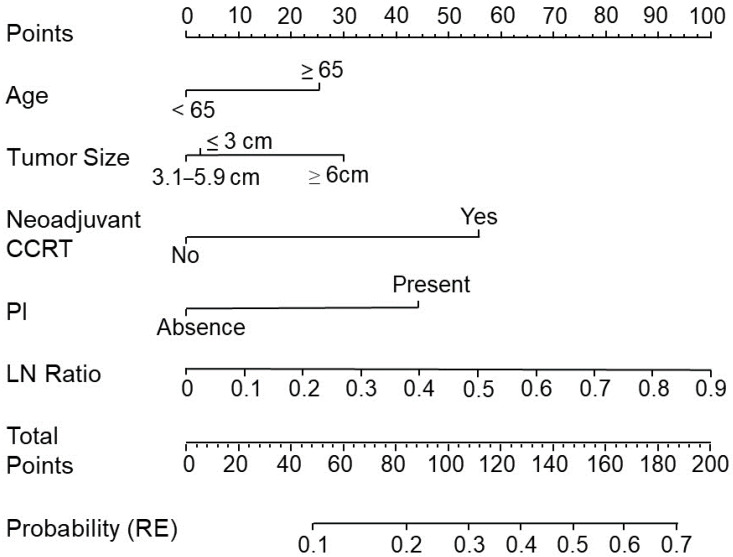
An established nomogram for predicting cancer recurrence. Nomogram incorporating clinical and pathological factors for predicting recurrence in patients with resected stage II–III CRC. There were five prognostic factors: age, tumor size, neoadjuvant chemoradiotherapy (CCRT), perineural invasion (PNI), and lymph node ratio (LNR). A straight line was drawn up to the points axis to determine how many points were associated with recurrence (RE). This process was repeated for each prognostic factor. The total points received for each prognostic factor are shown. From this, we calculated the probability of RE.

To evaluate the performance of the ML models, the AUCs for LR (AUC = 0.678), RF (AUC = 0.639), CART (AUC = 0.593), and SVM (AUC = 0.581) are shown in Figure 1. In the testing datasets, the accuracies of LR, RF, CART, and SVM were 0.87, 0.84, 0.83, and 0.86, respectively. According to the mean decrease in the impurity of the RF model, eight leading variables (mean decrease in accuracy > 3) were identified to be correlated with tumor recurrence and included high LNR, age, pT4 tumor invasion stage, high tumor volume, tumor site, lymphovascular invasion, tumor size, and chemotherapy (Figure 2A). According to the CART decision tree algorithm, LVI, PNI, age ≥ 63 years, lymph node ratio ≥ 0.12, and tumor volume ≥ 17 cm^3^ (Figure 2B) were the selected features related to tumor recurrence. The number 128/859 at the top node represents a total of 859 data points in the training set, among which there were 128 cases of cancer recurrence. The branch rule of this node was lymphovascular (LV) invasion is not identified (yes) or identified (no). In this, if LV invasion is not identified, the patient will be classified into the left node, while in the other result the patient will be classified to the node on the right. In the end, each patient will be classified as having cancer recurrence or not having cancer recurrence by this model. Among all the examining models, the LR model with a nomogram demonstrated a superior accuracy value of 0.87 and a specificity value of 1 in the testing set. The nomogram prediction model for cancer recurrence, with a recurrence probability ranging from 0.1 to 0.7, showed that age ≥ 65 years, tumor size ≥ 6 cm, high LNR, PNI, and neoadjuvant CCRT were significant variables associated with cancer recurrence (Figure 3). The value of 0.15 might be the best cutoff score for “protection” and the “risk” of recurrence by the Youden index (Supplementary Table S1) [22]. To identify significant risk factors, the prediction performances of clinicopathological variables selected by all ML models were evaluated using an ensemble-based voting system [23]. The results of ensemble voting for all identified risk factors for cancer recurrence are presented in Supplementary Table S2. Among all selected features, age and LNR were the only two factors identified by all ML models.

### 3.3. Different Clinicopathological Features between Younger and Older Patients with CRC

Although several studies have shown that age is a prognostic factor for the survival of patients with CRC, the impact of age on tumor recurrence for stage II and III disease is yet to be clearly elucidated [24,25,26]. To evaluate the predictive value of age for tumor recurrence, we used Cox proportional hazards models for multivariable analyses of patients with CRC in this study. In addition to tumor size, LNR, PNI, and neoadjuvant CCRT, multivariable analyses demonstrated that age ≥ 65 years was an independent risk factor for cancer recurrence and RFS (Table 2 and Table 3), in which these variables were previously identified through either the ML models or conventional statistics. We also used ML models for recurrence risk prediction, since ML algorithms are designed to handle complex features and non-linear properties. Although tumor size was not identified to be correlated with recurrence by ML algo-rithms, in previous studies, tumor size was indeed indicated as an adverse prognostic factor and was able to improve the performance of prognostic prediction for colorectal cancer [27]. In fact, when extreme spectrum of age was taken into consideration by conventional statistical analysis, i.e., when clinicopathological features between younger (age ≤ 50 years) and older (age ≥ 70 years) patients with stage II–III CRC were compared as shown in Table 4, older patients tended to have larger tumor size (*p* = 0.008) and increased tumor volume (*p* = 0.049), compared to the younger counterparts. Finally, comparing to the younger patients, the older patients were less frequently administered chemotherapy (*p* < 0.001), radiotherapy (*p* < 0.001), and neoadjuvant CCRT (*p* < 0.001). 

**Figure 4:**
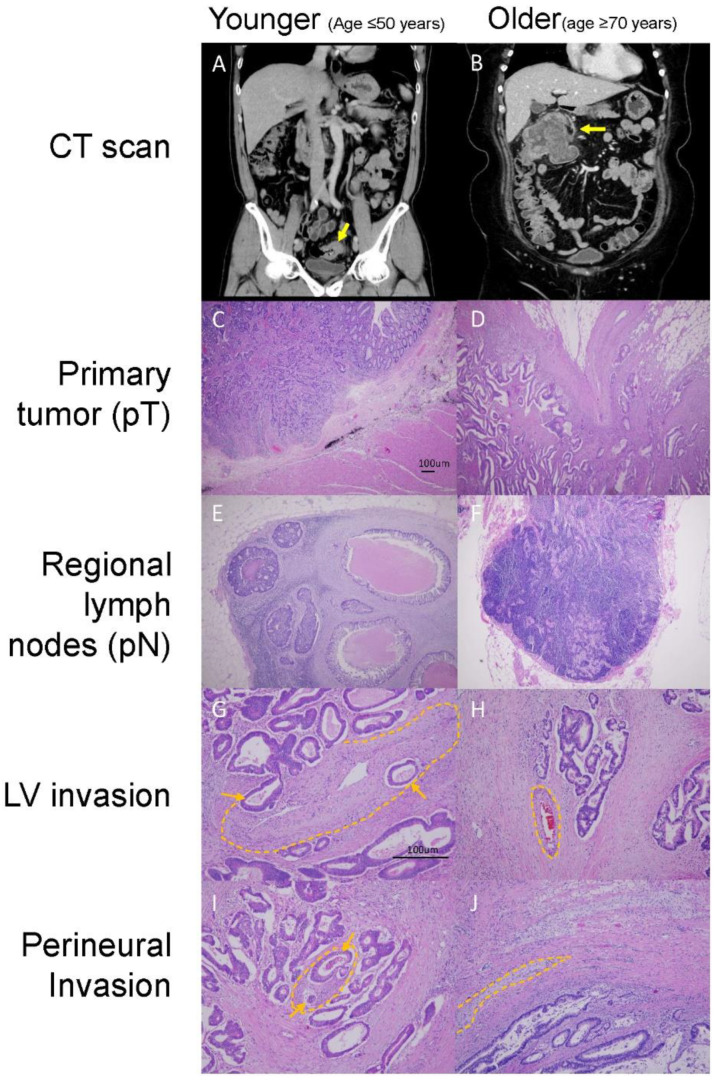
An example of younger (age ≤ 50 years) and older (age ≥ 70 years) patients with distinct clinicopathological features present in computed tomography (CT) scans and tumor tissues stained with hematoxylin-eosin. CT scans of a (**A**) younger patient with sigmoid cancer with a size <3 cm and (**B**) an older patient with hepatic flexure colon cancer with a size ≥6 cm. Tumor tissues with (**C**) low-depth tumor invasion stage (pT1, 40×), (**D**) high-depth tumor invasion stage (pT3, 40×), (**E**) positivity for lymph node (LN) metastasis (40×) (**F**), no LN metastasis (40×), (**G**) positivity for lymphovascular (LV) invasion (100×), (**H**) no lymphovascular invasion (100×), (**I**) positivity for perineural invasion (100×), and (**J**) no perineural invasion (100×) are shown. Orange arrows and dotted lines highlight the invasive tumor cells in the vessels and nerves in the figure (**G**,**I**). The vessels and nerves in (**H**,**J**) (highlighted by dotted lines) are negative for tumor involvement.

To present typical clinical scenarios, we provided an exemplary clinical presentation to illustrate common differences between younger and older stage II and III CRC patients, as shown in Figure 4. The older patients tended to have more right-sided colon tumors (*p* = 0.002), larger tumor sizes (*p* = 0.008), increased tumor volumes (*p* = 0.049), and more advanced disease in terms of pT stage (pT3 + pT4). However, the number of lymph node metastases (*p* = 0.001), LNRs (*p* = 0.004), and PNIs (*p* = 0.018) tended to be lower in older patients. Most importantly, the survival rates significantly decreased (*p* < 0.001) in older patients compared to younger patients. These results suggest that there are distinct clinicopathological behaviors of CRC tumors in older patients.

### 3.4. The Genomic Landscape in Older Patients with CRC

**Figure 5:**
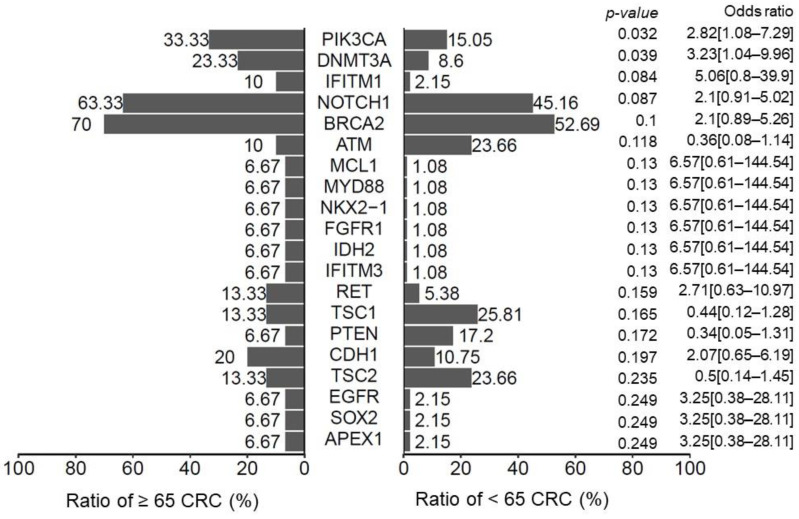
Histogram showing the percentage of genetic mutations in patients with CRC. We divided the patients into two subgroups: age ≥ 65 years and age < 65 years. The *p*-value was calculated using the Fisher exact test. In total, 30 (24.3%) patients were older than 65 years and *PIK3CA* and *DNMT3A* mutations were more prominent in these patients.

Cancer is a genomic disease, and genomic alterations on pivotal signaling pathways can regulate the growth and survival of tumor cells. To explore the molecular mechanisms underlying distinct clinicopathological features that are affected by aging, we investigated the genomic background of CRC tumors using a targeted-gene sequencing technique. According to genomic datasets derived from 123 CRC tumor specimens in this study (Figure 5), the number of *PIK3CA* and *DNMT3A* mutations was significantly increased in patients aged over 65 years (*p* = 0.032 and 0.039, respectively). Moreover, the genomic spectrum of *PIK3CA* also differed between older and younger patients with stage II–III CRC (Supplementary Table S3). Among the most prevalent *PIK3CA* variants, four p.E545K and two p.H1047R mutations were noted in the group of younger patients; however, only one p.E542K and p.H1047R mutation was found in the group of older patients. Most *PIK3CA* mutations were located at exons 5, 14, and 21 in older patients with CRC. These results imply that genetic backgrounds may be correlated with increased cancer recurrence associated with aging. All clinical features with genetic variants can be found in Supplementary Tables S4–S6. 

## 4. Discussion

In this study, we explored the value of ML models in predicting cancer recurrence for patients with stage II–III CRC after surgery by analyzing the clinicopathological and histopathological features from medical records and standardized pathology reports, respectively. For clinical application, we constructed a nomogram for predicting cancer recurrence with a high accuracy. Our findings revealed age >65 years at the time of initial diagnosis and high LNR to be important factors in predicting the tumor recurrence and survival outcomes of patients with stage II–III CRC. The panel-targeted sequencing results of tumor specimens revealed high incidences of oncogenic *PIK3CA* and *DNMT3A* mutations in patients aged ≥65 years, which highlighted the impact of age-dependent genomic alterations on CRC tumorigenesis. These results demonstrate the potential of integrating analyses of basic clinicopathological features and genomic analyses in order to create better predictive tools for assessing cancer recurrence.

To date, several studies have reported the promising performance of ML models in predicting the tumor recurrence and clinical survival outcomes of patients with CRC [14,15,28,29]. In previous reports investigating ML performance, patients with all stages of CRC have been included. The present study is the first to examine the predictive performance of ML algorithms specifically for patients with stage II–III CRC. Our studies showed the high and comparable performances of ML models in predicting tumor recurrence in stage II–III CRC, with the accuracies of the LR, RF, CART, and SVM models being 0.87, 0.84, 0.83, and 0.86 in the testing datasets, respectively. Several recurrence prediction nomograms based on LR models have been reported for patients with stage II–III CRC [30,31,32,33,34]. These nomograms can be pictorial representations of complex mathematical formulas with the primary advantages of estimating individualized risk based on histopathological features and patient characteristics [35]. However, the efficacies of the current recurrence prediction nomograms for stage II–III CRC may be restrained due to their retrospective nature and other analytical limitations [36]. Weiser [34] and Valentini [37] reported on two nomograms that had significant discriminative abilities with external validation for the OS of patients with stage II–III CRC. The respective AUCs of these two nomograms were 0.67 and 0.71; these values are comparable with the results from our LR model with the nomogram and training sets of ML models. Therefore, our prediction nomogram may be a useful tool for predicting recurrence in stage II and III CRC. The integration of a nomogram approach and ML models has been examined with regard to achieving a better data transparency and accuracy in predicting the clinical outcomes of patients with cancer [38]; further studies on stage II–III CRC are warranted.

In our study, LNR and age were found to be the two most significant risk factors for cancer recurrence, as determined by all four ML models (Supplementary Table S2). Some studies state that LNR is a significant risk factor that should be incorporated into the TNM staging system of the American Joint Committee on Cancer due to its high predictive value for survival [39,40]. However, some studies disagree with this because the lymph node assessment in CRC specimens can be influenced by both surgical and pathological factors, including the extent of the lymph node dissection, the length of the surgical specimen, the surgeon’s technique, and the thoroughness of the pathologists [41,42,43]. In our study, the mean number of total harvested lymph nodes was over 20, which is a proxy for high-quality surgical resection [44]. Under these circumstances, LNR emerged as a better discriminatory parameter in our specimens for predicting cancer recurrence [45]. The optimal cut-off point for LNR for cancer recurrence prediction was 0.12, as determined by the CART algorithm in our study. Comparable to studies examining LNR for cancer recurrence or survival risk stratification based on conventional statistical methods, our study may offer a perspective on objective risk stratification using LNR for stage II–III CRC patients.

The worse survival outcomes of older patients with stage II–III CRC are believed to be correlated with their higher levels of comorbidity, pre-existing conditions, and less frequent administration of adjuvant chemotherapy or radiotherapy [26,46,47,48]. To further explore the distinct biological behaviors correlated with the aging process, we compared clinicopathological features between younger and older patients with stage II–III CRC. For patients older than 70 years, the tumor sizes and volumes were significantly larger than those of patients younger than 50 years. It is reasonable to assume that larger tumor size is inversely correlated with survival in older CRC patients. Beyond our expectation, increased numbers of lymph node metastases and advanced pN stages were observed in younger patients compared to older patients. Due to their early pN stages, a lower utilization of adjuvant chemotherapy may be correlated with older patients with CRC [49]. Consistent with the real-world data from the National Cancer Database of the United States [6], the highest rate of adjuvant chemotherapy was observed among younger patients with high-risk histopathological features, whereas older patients without high-risk histopathological features had the lowest rate of adjuvant chemotherapy use. Therefore, our study demonstrates that distinct biological behaviors do indeed exist between younger and older stage II–III CRC patients and can influence the clinical decision-making process relating to the initiation of chemotherapy.

As distinct biological behaviors were observed between younger and older patients with CRC, genomic profiles were examined to explore the underlying molecular mechanisms. In our study, the mutation landscapes and signatures of older patients were significantly different from those seen in their younger counterparts. Although defective mismatch repair statuses and *BRAF* mutations were found to be statistically similar between the different age groups, the number of *PIK3CA* and *NDMT3A* mutations was significantly increased in patients with CRC aged over 65 years. It has been reported that *PIK3CA* mutations are present in 10–20% of CRC cases. In a previous study, *PIK3CA* genomic variants were associated with a higher TNM staging, and *PIK3CA* mutations were shown to confer resistance to first-line chemotherapy in patients with CRC [50]. The mutated loci were mainly located on exon 10 (E545K, E542K, and E545D) and exon 21 (H1047R). In our study, the mutation frequency of the *PIK3CA* gene in older CRC patients was 33.3%, which was higher than that recorded in their younger counterparts. Most young patients carried the E545K mutation, while most older patients had the E542K mutation. In our study, most older patients had right-sided colon cancer. From right- to left-sided colon cancer, a gradual decrease in *PIK3CA* mutation rates from as high as 21–25% down to 8–9% has been observed in the literature [51]. Furthermore, *DNMT3A* is a key player in DNA methylation, which plays an important role in multistage carcinogenesis [52]. Several studies have demonstrated that DNA methylation statuses can predict the therapeutic outcomes of patients with CRC [53]. Our genomic studies provide biological evidence that the aging process determines the disparate clinical behaviors and survival outcomes of patents with CRC. The potential benefits of harmonizing genetic information in cancer recurrence prediction models have been demonstrated for patients with CRC [54,55]. Our results support the importance of molecular and genomic profiling for predicting cancer recurrence in patients with stage II–III CRC after surgery.

The current study has some significant limitations. First, many confounding factors could impact the results. The comorbidities of patients were not included as covariates. As a result, survival analyses could not be performed when comorbidity and high-risk histopathologic features were taken into consideration. Moreover, the heterogenicity of treatment modality of patients with CRC (e.g., adjuvant chemotherapy and neoadjuvant radiotherapy) may be the potential biases in this study. Notably, patients with defective mismatch repair (dMMR) CRC, whose tumors are characterized by high-level microsatellite instability (MSI-H), have distinct clinical characteristics [56,57,58,59,60]. Based on several clinical studies, patients with stage II or III dMMR CRC have superior survival outcomes and lack benefits from adjuvant chemotherapy with 5-fluorouracil alone. Because of confounding effects from notable clinicopathological factors, no significant survival difference was observed in patients with disparate MMR expression in the present study. However, the study goal was to utilize standardized pathology reports and medical records to estimate the risk of cancer recurrence in our patients. By combining multivariable analysis, nomogram risk evaluation, and ML models, we have provided a useful clinical model that will help surgeons and oncologists to make better-informed decisions regarding their use of adjuvant chemotherapy and in future follow-ups. Next, the clinical characteristics of untreated—i.e., given no adjuvant chemotherapy—CRC stage II patients (N = 93) are presented in Supplementary Table S7. In CRC tissues, tumor budding is histologically defined by a single tumor cell or a small cluster of fewer than five tumor cells separated from the main tumor and present at the invasive front [61]. This unique histological manifestation is believed to be the biological representation of invasion initiation and metastasis cascade of CRC cells. Accumulation evidence has shown that the presence of tumor budding is a prognostic factor for inferior survivals of patients with CRC [62,63]. Comparable to previous studies, tumor budding was significantly associated with tumor recurrence (as shown in Table 1; *p* = 0.004) by conventional statistical analyses in this study. Since the recurrence numbers of patients with stage II CRC were limited, the clinical utility of tumor budding, as a prognostic factor, could not be shown in this group of patients.

Moreover, according to the International Tumor Budding Consensus Conference 2016, three different budding grades are classified as follows: Bd1 (0–4 buds/0.785 mm^2^), Bd2 (5–9 buds/0.785 mm^2^), and Bd3 (10 or more buds/0.785 mm^2^) [61]. Some studies have demonstrated that the high grade of tumor budding is an independent prognostic factor for shorter survivals of patients with stage II CRC [61]. In Taiwan, because tumor budding grades are not required in pathologic reports of CRC tissues, the clinical impact of these pathologic grades on patients is lacking in this study. The low rates of recurrence in untreated stage II patients coincide with the results of previous studies, demonstrating that the benefits of administering adjuvant chemotherapy in lower-risk stage II patients are low. Furthermore, we did not mention the y-prefix in cases with neoadjuvant treatment, since there were missing data for ypT cases (n = 2) and ypN cases (n = 24). There were a total of 67 ypStage II cases and 53 ypStage III cases, and these limited y-prefix case numbers should not have a significant statistical impact on our results, as demonstrated in Supplementary Table S8.

Second, a total of 1073 CRC patients were enrolled in this study. One hundred and fifty-nine (14.8%) patients developed cancer recurrence, while 914 (85.2%) patients did not. Considering that the ratio of these two groups was relatively uneven, we used stratified five-fold cross-validation to maintain the data characteristics. The distribution of the sample sizes could influence the performance of machine learning models. In this study, we created machine learning methods for risk factor analyses in the future. The best prediction power for cancer recurrence was achieved by the application of the LR model for analyzing our testing sets. Even though LR with nomogram (linear analysis) and ML (non-linear analysis) are different, similar results between different analytic models should be expected when a standardized pathology report and fixed sets of clinical characteristics are used for the analysis of stage II–III CRC. In other words, we should not expect the ML models to have a dramatically better predictive power than the LR models with nomograms if exactly the same variables are analyzed by both methods. Instead, more attention should be focused on the lack of complete molecular and genomic profiles of patients and the inadequacy of current clinicopathological information for predicting cancer recurrence in stage II–III CRC patients [25]. In other words, to increase the predictive power of both the nomogram and ML models, complete molecular and genomic profiles, as routine variables in standardized pathology reports, should be used in the future. However, our genomic results should be interpreted with caution because whole-genome analyses were performed in only 11% of the study population.

## 5. Conclusions

In summary, we demonstrated that ML models have a comparable predictive power for assessing cancer recurrence in patients with stage II–III CRC after surgery. We also built a prediction nomogram model for tumor recurrence. Advanced age and high LNR are significant risk factors for tumor recurrence. Age-associated genomic profiles may partly contribute to the distinct clinical behaviors and survival outcomes of patients with CRC. Studies incorporating complete molecular and genomic profiles into their cancer prediction models for these patients are warranted.

## Figures and Tables

**Table 1:** Characteristics of patients with stage II and III colorectal cancer.

Characteristic	Overall (N = 1073)	Recurrence		*p*-Value
		Yes (*n* = 159)	No (*n* = 914)	
Sex				0.062
Male	616 (57.4%)	102 (64.2%)	514 (56.2%)	
Female	457 (42.6%)	57 (35.8%)	400 (43.8%)	
Age (years)	65.39 (12.88)	65.86 (12.97)	65.31 (12.87)	0.432
BMI	23.80 (3.70)	23.87 (3.79)	23.78 (3.69)	0.992
Radiotherapy				0.288
No	786 (73.3%)	111 (69.8%)	675 (73.9%)	
Yes	287 (26.7%)	48 (30.2%)	239 (26.1%)	
Chemotherapy				0.003
No	138 (12.9%)	9 (5.7%)	129 (14.1%)	
Yes	935 (87.1%)	150 (94.3%)	785 (85.9%)	
Tumor site				0.466
Right side colon	322 (30.3%)	42 (26.6%)	280 (30.9%)	
Left side colon	464 (43.7%)	70 (44.3%)	394 (43.5%)	
Rectum	277 (26.1%)	46 (29.1%)	231 (25.5%)	
Tumor configuration				0.008
Endophytic	733 (69.3%)	123 (78.3%)	610 (67.8%)	
Exophytic	324 (30.7%)	34 (21.7%)	290 (32.2%)	
Tumor size				0.189
≤3 cm	270 (25.9%)	39 (25.3%)	231 (26.0%)	
3.1–5.9 cm	489 (46.8%)	64 (41.6%)	425 (47.8%)	
≥6 cm	285 (27.3%)	51 (33.1%)	234 (26.3%)	
Tumor volume	42.13 (111.09)	59.89 (148.86)	39.09 (103.07)	0.066
Histologic grade				0.084
Poorly diff.	48 (4.6%)	11 (7.1%)	37 (4.2%)	
Moderately diff.	868 (83.0%)	132 (84.6%)	736 (82.7%)	
Well-diff.	130 (12.4%)	13 (8.3%)	117 (13.1%)	
TNM stage *				<0.001
II	528 (49.2%)	56 (35.2%)	472 (51.6%)	
III	545 (50.8%)	103 (64.8%)	442 (48.4%)	
Tumor invasion stage (pT)				<0.001
1–2	75 (7.0%)	6 (3.8%)	69 (7.6%)	
3	858 (80.6%)	115 (73.2%)	743 (81.9%)	
4	131 (12.3%)	36 (22.9%)	95 (10.5%)	
Lymph nodes status (pN)				<0.001
0	488 (49.3%)	53 (35.3%)	435 (51.8%)	
1	343 (34.7%)	54 (36.0%)	289 (34.4%)	
2	158 (16.0%)	43 (28.7%)	115 (13.7%)	
LN metastasis numbers	1.74 (3.37)	3.16 (4.58)	1.50 (3.05)	<0.001
Total harvested LNs	20.02 (9.26)	19.35 (8.35)	20.14 (9.41)	0.749
LN ratio	0.09 (0.15)	0.16 (0.21)	0.08 (0.14)	<0.001
Distal margin				0.159
Uninvolved	1061 (99.5%)	155 (98.7%)	906 (99.7%)	
Involved	5 (0.5%)	2 (1.3%)	3 (0.3%)	
Circumferential margin				0.075
Uninvolved	309 (91.2%)	56 (90.3%)	253 (91.3%)	
Involved	13 (3.8%)	5 (8.1%)	8 (2.9%)	
Cannot be assessed	17 (5.0%)	1 (1.6%)	16 (5.8%)	
Lymphovascular invasion				<0.001
Absence	624 (58.2%)	57 (35.8%)	567 (62.1%)	
Present	448 (41.8%)	102 (64.2%)	346 (37.9%)	
Perineural invasion				<0.001
Absence	571 (53.3%)	53 (33.3%)	518 (56.7%)	
Present	501 (46.7%)	106 (66.7%)	395 (43.3%)	
Tumor growth pattern				0.083
Infiltrating	969 (90.6%)	150 (94.3%)	819 (90.0%)	
Pushing	100 (9.4%)	9 (5.7%)	91 (10.0%)	
Tumor budding				0.004
Absent	342 (81.4%)	20 (62.5%)	322 (83%)	
Present	78 (18.6%)	12 (37.5%)	66 (17%)	
TRG				0.312
1–2	15 (12.5%)	5 (19.2%)	10 (10.6%)	
3–5	105 (87.5%)	21 (80.8%)	84 (89.4%)	
Neoadjuvant CCRT				0.025
No	953 (88.8%)	133 (83.6%)	820 (89.7%)	
Yes	120 (11.2%)	26 (16.4%)	94 (10.3%)	
dMMR				0.932
Deficient	35 (10.1%)	3 (9.7%)	32 (10.2%)	
Proficient	311 (89.9%)	28 (90.3%)	283 (89.8%)	
BRAF_V600E_Stain				>0.999
Negative	101 (85.6%)	12 (85.7%)	89 (85.6%)	
Positive	17 (14.4%)	2 (14.3%)	15 (14.4%)	
Alive				<0.001
Yes	912 (85.0%)	86 (54.1%)	826 (90.4%)	
No	161 (15.0%)	73 (45.9%)	88 (9.6%)	

* The American Joint Committee on Cancer Stage System. All patients had no metastasis at the initial diagnosis (M0). ypStage II (*n* = 67) is grouped as Stage II, ypStage III (*n* = 53) is grouped as Stage III. Abbreviations: BMI, body mass index; diff, differentiated; pT, depth of tumor invasion stage; pN, lymph node stage; LN, lymph node; TRG, tumor regression grade; CCRT, chemoradiotherapy; dMMR, defective mismatch repair.

**Table 2:** Univariable and multivariable analyses of variables associated with the probability of cancer recurrence.

Characteristic *	Univariable Analysis			Multivariable Analysis		
	OR	95% CI	*p*-Value	OR	95% CI	*p*-Value
Age (years)						
<65 vs. ≥65	1.24	0.85–1.81	0.273	1.70	1.12–2.61	0.014
Tumor size						
1 vs. 2 (3.1–5.9 cm)	0.88	0.55–1.44	0.612	0.94	0.56–1.60	0.808
1 vs. 3 (≥6 cm)	1.21	0.73–2.03	0.466	1.79	1.01–3.22	0.047
Lymph node ratio	16.9	5.93–48.1	<0.001	10.8	3.51–33.2	<0.001
Perineural invasion						
No vs. Present	2.37	1.61–3.52	<0.001	2.51	1.63–3.91	<0.001
Neoadjuvant CCRT						
No vs. Yes	2.12	1.25–3.47	0.004	3.34	1.80–6.12	<0.001

Abbreviations: OR, odds ratio; CI, confidence interval; vs., versus; 1, ≤3.0 cm; 2, 3.1–5.9 cm; 3, ≥6 cm; CCRT, chemoradiotherapy. * The variables were previously identified through either the ML models or conventional statistical analyses.

**Table 3:** Univariable and multivariable analyses of variables associated with recurrence-free survival.

Characteristic *	Univariable Analysis			Multivariable Analysis		
	HR	95% CI	*p*-Value	HR	95% CI	*p*-Value
Age (years)						
<65 vs. ≥65	1.25	0.91–1.71	0.164	1.74	1.24–2.44	0.001
Tumor size						
1 vs. 2 (3.1–5.9 cm)	0.87	0.59–1.30	0.500	0.92	0.61–1.41	0.711
1 vs. 3 (≥6 cm)	1.19	0.79–1.81	0.406	1.82	1.16–2.85	0.010
Lymph node ratio	10.1	4.95–20.6	<0.001	6.82	3.20–14.5	<0.001
Perineural invasion						
No vs. Present	2.73	1.96–3.80	<0.001	2.90	2.03–4.16	<0.001
Neoadjuvant CCRT						
No vs. Yes	1.68	1.10–2.55	0.016	2.35	1.46–3.81	<0.001

Abbreviations: HR, hazard ratio; CI, confidence interval; vs., versus; 1, ≤3.0 cm; 2, 3.1–5.9 cm; 3, ≥6 cm; CCRT, chemoradiotherapy. * The variables were previously identified through either the ML models or conventional statistical analyses.

**Table 4:** Characteristics of patients with stage II and III colorectal cancer.

Characteristic	Age (Years)			*p*-Value
	≤50 (*n* = 150)	51–69 (*n* = 470)	≥70 (*n* = 453)	
Sex				0.003
Male	80 (53.3%)	297 (63.2%)	239 (52.8%)	
Female	70 (46.7%)	173 (36.8%)	214 (47.2%)	
Age (years)	43.47 (5.81)	60.72 (5.20)	77.49 (5.02)	<0.001
BMI	24.00 (3.94)	24.03 (3.77)	23.49 (3.52)	0.206
Radiotherapy				<0.001
No	97 (64.7%)	314 (66.8%)	375 (82.8%)	
Yes	53 (35.3%)	156 (33.2%)	78 (17.2%)	
Chemotherapy				<0.001
No	10 (6.7%)	33 (7.0%)	95 (21.0%)	
Yes	140 (93.3%)	437 (93.0%)	358 (79.0%)	
Tumor site				0.002
Right side colon	37 (24.7%)	126 (26.9%)	159 (35.7%)	
Left side colon	63 (42.0%)	208 (44.4%)	193 (43.4%)	
Rectum	50 (33.3%)	134 (28.6%)	93 (20.9%)	
Tumor configuration				0.549
Endophytic	113 (75.3%)	322 (69.4%)	298 (67.3%)	
Exophytic	37 (24.7%)	142 (30.6%)	145 (32.7%)	
Tumor size				0.008
≤3 cm	42 (28.6%)	140 (30.3%)	88 (20.2%)	
3.1–5.9 cm	70 (47.6%)	207 (44.8%)	212 (48.7%)	
≥6 cm	35 (23.8%)	115 (24.9%)	135 (31.0%)	
Tumor volume	38.80 (81.50)	35.99 (67.57)	49.62 (149.17)	0.049
Histology grade				0.812
Poorly diff.	9 (6.1%)	20 (4.4%)	19 (4.3%)	
Moderately diff.	124 (83.8%)	380 (82.8%)	364 (82.9%)	
Well-diff.	15 (10.1%)	59 (12.9%)	56 (12.8%)	
TNM stage *				<0.001
II	59 (39.3%)	217 (46.2%)	252 (55.6%)	
III	91 (60.7%)	253 (53.8%)	201 (44.4%)	
Tumor invasion stage (pT)				0.040
1–2	10 (6.8%)	44 (9.5%)	21 (4.6%)	
3	115 (77.7%)	369 (79.5%)	374 (82.7%)	
4	23 (15.5%)	51 (11.0%)	57 (12.6%)	
LN status (pN)				0.005
0	53 (40.2%)	197 (45.7%)	238 (55.9%)	
1	54 (40.9%)	156 (36.2%)	133 (31.2%)	
2	25 (18.9%)	78 (18.1%)	55 (12.9%)	
LN metastasis numbers	2.26 (4.05)	1.80 (3.03)	1.51 (3.44)	0.001
Total harvested LNs	21.62 (10.40)	19.89 (9.54)	19.63 (8.49)	0.207
LN ratio	0.10 (0.16)	0.10 (0.16)	0.08 (0.14)	0.004
Distal margin				>0.999
Uninvolved	149 (100.0%)	466 (99.4%)	446 (99.6%)	
Involved	0 (0.0%)	3 (0.6%)	2 (0.4%)	
Circumferential margin				0.412
Uninvolved	54 (96.4%)	140 (88.1%)	115 (92.7%)	
Involved	1 (1.8%)	9 (5.7%)	3 (2.4%)	
Cannot be assessed	1 (1.8%)	10 (6.3%)	6 (4.8%)	
Lymphovascular invasion				0.212
Absence	78 (52.0%)	274 (58.3%)	272 (60.2%)	
Present	72 (48.0%)	196 (41.7%)	180 (39.8%)	
Perineural invasion				0.018
Absence	65 (43.3%)	250 (53.2%)	256 (56.6%)	
Present	85 (56.7%)	220 (46.8%)	196 (43.4%)	
Growth pattern at tumor periphery				0.273
Infiltrating	141 (94.0%)	424 (90.6%)	404 (89.6%)	
Pushing	9 (6.0%)	44 (9.4%)	47 (10.4%)	
Tumor budding				0.568
Absence	59 (83.1%)	144 (79.1%)	139 (83.2%)	
Present	12 (16.9%)	38 (20.9%)	28 (16.8%)	
TRG				0.765
1–2	5 (16.1%)	7 (11.5%)	3 (10.7%)	
3–5	26 (83.9%)	54 (88.5%)	25 (89.3%)	
Neoadjuvant CCRT				<0.001
No	119 (79.3%)	409 (87.0%)	425 (93.8%)	
Yes	31 (20.7%)	61 (13.0%)	28 (6.2%)	
dMMR				0.488
Deficient	9 (14.3%)	14 (9.2%)	12 (9.2%)	
Proficient	54 (85.7%)	138 (90.8%)	119 (90.8%)	
BRAF_V600E_stain				0.076
Negative	23 (100.0%)	42 (82.4%)	36 (81.8%)	
Positive	0 (0.0%)	9 (17.6%)	8 (18.2%)	
Recurrence				0.698
No	129 (86.0%)	404 (86.0%)	381 (84.1%)	
Yes	21 (14.0%)	66 (14.0%)	72 (15.9%)	
Alive				<0.001
Yes	137 (91.3%)	425 (90.4%)	350 (77.3%)	
No	13 (8.7%)	45 (9.6%)	103 (22.7%)	

* The American Joint Committee on Cancer Stage System. All patients had no metastasis at the initial diagnosis (M0). ypStage II (*n* = 67) is grouped as Stage II, ypStage III (*n* = 53) is grouped as Stage III. Abbreviations: BMI: body mass index; differentiated, diff; pT: depth of tumor invasion stage; pN: lymph node stage; LN: lymph node; TRG: tumor regression grade; CCRT: chemoradiotherapy; dMMR; defective mismatch repair.

## Data Availability

The datasets used and analyzed during the current study are available from the corresponding author on reasonable request, and supplementary information files are available for this manuscript.

## Supplementary Materials

The following supporting information can be downloaded at: https://www.mdpi.com/article/10.3390/biomedicines10020340/s1, Table S1: Optimal Cutoff Score by Youden Index; Table S2: Risk Factors; Table S3: PIK3CA Gene Mutations; Table S4: Oncomine Cancer Gene Mutation Panel; Table S5: Clinical features of 123 patient with cancer gene panel; Table S6: Genetic Variatns in 123 Cancer Patients with OCP; Table S7: Untreated Stage II Colorectal Cancer Patients; Table S8: Neoadjuvant Chemoradiotherapy.

## References

[1] Sung H., Ferlay J., Siegel R.L., Laversanne M., Soerjomataram I., Jemal A., Bray F. (2021). Global Cancer Statistics 2020: GLOBOCAN Estimates of Incidence and Mortality Worldwide for 36 Cancers in 185 Countries. CA Cancer J. Clin..

[2] (2020). HPA. https://www.hpa.gov.tw/Pages/Detail.aspx?nodeid=269&pid=13498.

[3] Osterman E., Glimelius B. (2018). Recurrence Risk after Up-to-Date Colon Cancer Staging, Surgery, and Pathology: Analysis of the Entire Swedish Population. Dis. Colon Rectum.

[4] Osterman E., Hammarström K., Imam I., Osterlund E., Sjöblom T., Glimelius B. (2020). Recurrence Risk after Radical Colorectal Cancer Surgery—Less Than before, but How High Is It?. Cancers.

[5] Benson A.B., Venook A.P., Al-Hawary M.M., Arain M.A., Chen Y.-J., Ciombor K.K., Cohen S., Cooper H.S., Deming D., Farkas L. (2021). Colon Cancer, Version 2.2021, NCCN Clinical Practice Guidelines in Oncology. J. Natl. Compr. Cancer Netw..

[6] Enofe N., Morris A.D., Liu Y., Liang W., Wu C.S., Sullivan P.S., Balch G.G., Staley C.A., Gillespie T.W., Shaffer V.O. (2020). Receipt of Adjuvant Chemotherapy in Stage II Colon Cancer and Overall Survival: A National Cancer Database Study. J. Surg. Res..

[7] Papamichael D., Renfro L.A., Matthaiou C., Yothers G., Saltz L., Guthrie K.A., Van Cutsem E., Schmoll H.J., Labianca R., André T. (2016). Validity of Adjuvant! Online in older patients with stage III colon cancer based on 2967 patients from the ACCENT database. J. Geriatr. Oncol..

[8] Xu W., He Y., Wang Y., Li X., Young J., Ioannidis J.P.A., Dunlop M.G., Theodoratou E. (2020). Risk factors and risk prediction models for colorectal cancer metastasis and recurrence: An umbrella review of systematic reviews and meta-analyses of observational studies. BMC Med..

[9] Kim B.-H., Kim J.M., Kang G.H., Chang H.J., Kang D.W., Kim J.H., Bae J.M., Seo A.N., Park H.S., Kang Y.K. (2020). Standardized Pathology Report for Colorectal Cancer, 2nd Edition. J. Pathol. Transl. Med..

[10] Sluijter C.E., van Workum F., Wiggers T., van de Water C., Visser O., van Slooten H.J., Overbeek L.I.H., Nagtegaal I.D. (2019). Improvement of Care in Patients with Colorectal Cancer: Influence of the Introduction of Standardized Structured Reporting for Pathology. JCO Clin. Cancer Inform..

[11] Konishi T., Shimada Y., Hsu M., Wei I.H., Pappou E., Smith J.J., Nash G.M., Guillem J.G., Paty P.B., Garcia-Aguilar J. (2019). Contemporary Validation of a Nomogram Predicting Colon Cancer Recurrence, Revealing All-Stage Improved Outcomes. JNCI Cancer Spectr..

[12] He Y., Ong Y., Li X., Din F.V.N., Brown E., Timofeeva M., Wang Z., Farrington S.M., Campbell H., Dunlop M.G. (2019). Performance of prediction models on survival outcomes of colorectal cancer with surgical resection: A systematic review and meta-analysis. Surg. Oncol..

[13] Mitsala A., Tsalikidis C., Pitiakoudis M., Simopoulos C., Tsaroucha A.K. (2021). Artificial Intelligence in Colorectal Cancer Screening, Diagnosis and Treatment. A New Era. Curr. Oncol..

[14] Ting W.C., Lu Y.A., Ho W.C., Cheewakriangkrai C., Chang H.R., Lin C.L. (2020). Machine Learning in Prediction of Second Primary Cancer and Recurrence in Colorectal Cancer. Int. J. Med. Sci..

[15] Achilonu O.J., Fabian J., Bebington B., Singh E., Eijkemans M.J.C., Musenge E. (2021). Predicting Colorectal Cancer Recurrence and Patient Survival Using Supervised Machine Learning Approach: A South African Population-Based Study. Front. Public Health.

[16] Kourou K., Exarchos T.P., Exarchos K.P., Karamouzis M.V., Fotiadis D.I. (2015). Machine learning applications in cancer prognosis and prediction. Comput. Struct. Biotechnol. J..

[17] Irizarry R.A. (2019). Introduction to Data Science: Data Analysis and Prediction Algorithms with R.

[18] R Core Team (2020). R: A Language and Environment for Statistical Computing. R Foundation for Statistical Computing.

[19] Kattan M.W. (2002). Nomograms. Introduction. Semin Urol. Oncol..

[20] Wang K., Li M., Hakonarson H. (2010). ANNOVAR: Functional annotation of genetic variants from next-generation sequencing data. Nucleic Acids Res..

[21] Tsai Y.Y., Lee W.J. (2021). An imagined future community: Taiwan Biobank, Taiwanese genome, and nation-building. BioSocieties.

[22] Fluss R., Faraggi D., Reiser B. (2005). Estimation of the Youden Index and its associated cutoff point. Biom. J..

[23] Polikar R. (2006). Ensemble based systems in decision making. IEEE Circuits Syst. Mag..

[24] McKay A., Donaleshen J., Helewa R.M., Park J., Wirtzfeld D., Hochman D., Singh H., Turner D. (2014). Does young age influence the prognosis of colorectal cancer: A population-based analysis. World J. Surg. Oncol..

[25] Bouvier A.M., Launoy G., Bouvier V., Rollot F., Manfredi S., Faivre J., Cottet V., Jooste V. (2015). Incidence and patterns of late recurrences in colon cancer patients. Int. J. Cancer.

[26] Zare-Bandamiri M., Fararouei M., Zohourinia S., Daneshi N., Dianatinasab M. (2017). Risk Factors Predicting Colorectal Cancer Recurrence Following Initial Treatment: A 5-Year Cohort Study. Asian Pac. J. Cancer Prev..

[27] Liang Y., Li Q., He D., Chen Y., Li J. (2021). Tumor size improves the accuracy of the prognostic prediction of T4a stage colon cancer. Sci. Rep..

[28] Gupta P., Chiang S.-F., Sahoo P.K., Mohapatra S.K., You J.-F., Onthoni D.D., Hung H.-Y., Chiang J.-M., Huang Y., Tsai W.-S. (2019). Prediction of Colon Cancer Stages and Survival Period with Machine Learning Approach. Cancers.

[29] Xu Y., Ju L., Tong J., Zhou C.-M., Yang J.-J. (2020). Machine Learning Algorithms for Predicting the Recurrence of Stage IV Colorectal Cancer After Tumor Resection. Sci. Rep..

[30] Tsikitis V.L., Larson D.W., Huebner M., Lohse C.M., Thompson P.A. (2014). Predictors of recurrence free survival for patients with stage II and III colon cancer. BMC Cancer.

[31] Hoshino N., Hasegawa S., Hida K., Kawada K., Ganeko R., Sugihara K., Sakai Y. (2016). Nomogram for predicting recurrence in stage II colorectal cancer. Acta Oncol..

[32] Renfro L.A., Grothey A., Xue Y., Saltz L.B., André T., Twelves C., Labianca R., Allegra C.J., Alberts S.R., Loprinzi C.L. (2014). ACCENT-based web calculators to predict recurrence and overall survival in stage III colon cancer. J. Natl. Cancer Inst..

[33] Saso K., Myoshi N., Fujino S., Takenaka Y., Takahashi Y., Nishimura J., Yasui M., Ohue M., Tokuoka M., Ide Y. (2018). A novel prognostic prediction model for recurrence in patients with stage II colon cancer after curative resection. Mol. Clin. Oncol..

[34] Weiser M.R., Landmann R.G., Kattan M.W., Gonen M., Shia J., Chou J., Paty P.B., Guillem J.G., Temple L.K., Schrag D. (2008). Individualized prediction of colon cancer recurrence using a nomogram. J. Clin. Oncol..

[35] Balachandran V.P., Gonen M., Smith J.J., DeMatteo R.P. (2015). Nomograms in oncology: More than meets the eye. Lancet Oncol..

[36] Lemini R., Attwood K., Pecenka S., Grego J., Spaulding A.C., Nurkin S., Colibaseanu D.T., Gabriel E. (2018). Stage II-III colon cancer: A comparison of survival calculators. J. Gastrointest. Oncol..

[37] Valentini V., van Stiphout R.G., Lammering G., Gambacorta M.A., Barba M.C., Bebenek M., Bonnetain F., Bosset J.F., Bujko K., Cionini L. (2011). Nomograms for predicting local recurrence, distant metastases, and overall survival for patients with locally advanced rectal cancer on the basis of European randomized clinical trials. J. Clin. Oncol..

[38] Alabi R.O., Mäkitie A.A., Pirinen M., Elmusrati M., Leivo I., Almangush A. (2021). Comparison of nomogram with machine learning techniques for prediction of overall survival in patients with tongue cancer. Int. J. Med. Inform..

[39] Hong K.D., Lee S.I., Moon H.Y. (2011). Lymph node ratio as determined by the 7th edition of the American Joint Committee on Cancer staging system predicts survival in stage III colon cancer. J. Surg. Oncol..

[40] Chin C.C., Wang J.Y., Yeh C.Y., Kuo Y.H., Huang W.S., Yeh C.H. (2009). Metastatic lymph node ratio is a more precise predictor of prognosis than number of lymph node metastases in stage III colon cancer. Int. J. Color. Dis..

[41] Jakob M.O., Guller U., Ochsner A., Oertli D., Zuber M., Viehl C.T. (2018). Lymph node ratio is inferior to pN-stage in predicting outcome in colon cancer patients with high numbers of analyzed lymph nodes. BMC Surg..

[42] Gleisner A.L., Mogal H., Dodson R., Efron J., Gearhart S., Wick E., Lidor A., Herman J.M., Pawlik T.M. (2013). Nodal status, number of lymph nodes examined, and lymph node ratio: What defines prognosis after resection of colon adenocarcinoma?. J. Am. Coll. Surg..

[43] Zanghì A., Cavallaro A., Lo Menzo E., Curella Botta S., Lo Bianco S., Di Vita M., Cardì F., Cappellani A. (2020). Is there a relationship between length of resection and lymph-node ratio in colorectal cancer?. Gastroenterol. Rep..

[44] Trepanier M., Erkan A., Kouyoumdjian A., Nassif G., Albert M., Monson J., Lee L. (2019). Examining the relationship between lymph node harvest and survival in patients undergoing colectomy for colon adenocarcinoma. Surgery.

[45] Zhang M.-R., Xie T.-H., Chi J.-L., Li Y., Yang L., Yu Y.-Y., Sun X.-F., Zhou Z.-G. (2016). Prognostic role of the lymph node ratio in node positive colorectal cancer: A meta-analysis. Oncotarget.

[46] Merchant S.J., Nanji S., Brennan K., Karim S., Patel S.V., Biagi J.J., Booth C.M. (2017). Management of stage III colon cancer in the elderly: Practice patterns and outcomes in the general population. Cancer.

[47] Pilleron S., Gower H., Janssen-Heijnen M., Signal V.C., Gurney J.K., Morris E.J., Cunningham R., Sarfati D. (2021). Patterns of age disparities in colon and lung cancer survival: A systematic narrative literature review. BMJ Open.

[48] Mima K., Kurashige J., Miyanari N., Morito A., Yumoto S., Matsumoto T., Kosumi K., Inoue M., Mizumoto T., Kubota T. (2020). Advanced Age Is a Risk Factor for Recurrence After Resection in Stage II Colorectal Cancer. In Vivo.

[49] Saha S., Shaik M., Johnston G., Saha S.K., Berbiglia L., Hicks M., Gernand J., Grewal S., Arora M., Wiese D. (2015). Tumor size predicts long-term survival in colon cancer: An analysis of the National Cancer Data Base. Am. J. Surg..

[50] Wang Q., Shi Y.-L., Zhou K., Wang L.-L., Yan Z.-X., Liu Y.-L., Xu L.-L., Zhao S.-W., Chu H.-L., Shi T.-T. (2018). PIK3CA mutations confer resistance to first-line chemotherapy in colorectal cancer. Cell Death Dis..

[51] Day F.L., Jorissen R.N., Lipton L., Mouradov D., Sakthianandeswaren A., Christie M., Li S., Tsui C., Tie J., Desai J. (2013). PIK3CA and PTEN gene and exon mutation-specific clinicopathologic and molecular associations in colorectal cancer. Clin. Cancer Res..

[52] Zhang J., Yang C., Wu C., Cui W., Wang L. (2020). DNA Methyltransferases in Cancer: Biology, Paradox, Aberrations, and Targeted Therapy. Cancers.

[53] Cervena K., Siskova A., Buchler T., Vodicka P., Vymetalkova V. (2020). Methylation-Based Therapies for Colorectal Cancer. Cells.

[54] Tian X., Zhu X., Yan T., Yu C., Shen C., Hu Y., Hong J., Chen H., Fang J.Y. (2017). Recurrence-associated gene signature optimizes recurrence-free survival prediction of colorectal cancer. Mol. Oncol..

[55] Peng J., Wang Z., Chen W., Ding Y., Wang H., Huang H., Huang W., Cai S. (2010). Integration of genetic signature and TNM staging system for predicting the relapse of locally advanced colorectal cancer. Int. J. Color. Dis..

[56] Lindor N.M., Burgart L.J., Leontovich O., Goldberg R.M., Cunningham J.M., Sargent D.J., Walsh-Vockley C., Petersen G.M., Walsh M.D., Leggett B.A. (2002). Immunohistochemistry versus microsatellite instability testing in phenotyping colorectal tumors. J. Clin. Oncol..

[57] Ribic C.M., Sargent D.J., Moore M.J., Thibodeau S.N., French A.J., Goldberg R.M., Hamilton S.R., Laurent-Puig P., Gryfe R., Shepherd L.E. (2003). Tumor microsatellite-instability status as a predictor of benefit from fluorouracil-based adjuvant chemotherapy for colon cancer. N. Engl. J. Med..

[58] Lanza G., Gafà R., Santini A., Maestri I., Guerzoni L., Cavazzini L. (2006). Immunohistochemical test for MLH1 and MSH2 expression predicts clinical outcome in stage II and III colorectal cancer patients. J. Clin. Oncol..

[59] Jover R., Zapater P., Castells A., Llor X., Andreu M., Cubiella J., Balaguer F., Sempere L., Xicola R.M., Bujanda L. (2009). The efficacy of adjuvant chemotherapy with 5-fluorouracil in colorectal cancer depends on the mismatch repair status. Eur. J. Cancer.

[60] Cohen R., Taieb J., Fiskum J., Yothers G., Goldberg R., Yoshino T., Alberts S., Allegra C., de Gramont A., Seitz J.F. (2021). Microsatellite Instability in Patients with Stage III Colon Cancer Receiving Fluoropyrimidine With or Without Oxaliplatin: An ACCENT Pooled Analysis of 12 Adjuvant Trials. J. Clin. Oncol..

[61] Lugli A., Kirsch R., Ajioka Y., Bosman F., Cathomas G., Dawson H., El Zimaity H., Fléjou J.F., Hansen T.P., Hartmann A. (2017). Recommendations for reporting tumor budding in colorectal cancer based on the International Tumor Budding Consensus Conference (ITBCC) 2016. Mod. Pathol..

[62] Van Wyk H.C., Park J.H., Edwards J., Horgan P.G., McMillan D.C., Going J.J. (2016). The relationship between tumour budding, the tumour microenvironment and survival in patients with primary operable colorectal cancer. Br. J. Cancer.

[63] Van Wyk H.C., Roseweir A., Alexander P., Park J.H., Horgan P.G., McMillan D.C., Edwards J. (2019). The Relationship Between Tumor Budding, Tumor Microenvironment, and Survival in Patients with Primary Operable Colorectal Cancer. Ann. Surg. Oncol..

