# Supplementary material for: A Prediction Model for Tumor Recurrence in Stage II–III Colorectal Cancer Patients: From a Machine Learning Model to Genomic Profiling
Source: Biomedicines. 2022 Feb 1;10(2):340. doi: 10.3390/biomedicines10020340 (PMC8961774; doi:10.3390/biomedicines10020340)
Supplement: Supplementary file 1 [file biomedicines-10-00340-s001.zip › Supplementary Table S1. Optimal Cutoff Score by Youden Index 1222V1.pdf]

Supplementary Table S1. Optimal Cutoff Score by Youden Index

Cutoff	Youden index
0.10	0.2665
0.15	0.3118
0.20	0.2138
0.25	0.0591
0.30	0.1380
0.35	0.1168
0.40	0.0634
0.45	0.0045
0.50	0.0100
0.55	0.0156
0.60	0.0211
0.65	0.0211
0.70	0.0267
