# Supplementary material for: A Prediction Model for Tumor Recurrence in Stage II–III Colorectal Cancer Patients: From a Machine Learning Model to Genomic Profiling
Source: Biomedicines. 2022 Feb 1;10(2):340. doi: 10.3390/biomedicines10020340 (PMC8961774; doi:10.3390/biomedicines10020340)
Supplement: Supplementary file 1 [file biomedicines-10-00340-s001.zip › Supplementary Table S2. Risk Factors 1222V1.pdf]

Supplementary Table S2. Prognostic factors for cancer recurrence in different machine learning models

	**pT**	**Tumor Volume**	**Age**	**Tumor Size**	**CCRT**	**PNI**	**Tumor Site**	**LNR**	**LVI**
LR			V	V	V	V		V	
RF	V	V	V				V	V	
CART		V	V			V		V	V

Abbreviations: pT: depth of tumor Invasion stage; CCRT: chemoradiotherapy; PNI: perineural invasion; LNR: lymph node ratio; LVI: lymphovascular invasion. LR: logistic regression; RF: Random forest; CART: classification and regression trees.
