# Supplementary material for: A Prediction Model for Tumor Recurrence in Stage II–III Colorectal Cancer Patients: From a Machine Learning Model to Genomic Profiling
Source: Biomedicines. 2022 Feb 1;10(2):340. doi: 10.3390/biomedicines10020340 (PMC8961774; doi:10.3390/biomedicines10020340)
Supplement: Supplementary file 1 [file biomedicines-10-00340-s001.zip › Supplementary Table S7. Untreated Stage II Colorectal Cancer Patients 1222V1.pdf]

Supplementary Table S7. Characteristics of Patients with Untreated Stage II Colorectal Cancer Patients

		Recurrence	
**Characteristic**	**Overall (N = 93)**	**Yes (n = 4)**	**No (n = 89)**
Sex			
Male	45 (48.4%)	1 (25.0%)	44 (49.4%)
Female	48 (51.6%)	3 (75.0%)	45 (50.6%)
Age(years)	74.71 (12.10)	67.50 (14.06)	75.03 (11.99)
BMI	23.34 (4.14)	24.72 (3.83)	23.27 (4.16)
Radiotherapy			
No	89 (95.7%)	4 (100.0%)	85 (95.5%)
Yes	4 (4.3%)	0 (0.0%)	4 (4.5%)
Tumor site			
Right side colon	45 (48.9%)	2 (50.0%)	43 (48.9%)
Left side colon	35 (38.0%)	1 (25.0%)	34 (38.6%)
Rectum	12 (13.0%)	1 (25.0%)	11 (12.5%)
Tumor configuration			
Endophytic	49 (52.2%)	3 (75.0%)	45 (51.1%)
Exophytic	44 (47.8%)	1 (25.0%)	43 (48.9%)
Tumor size			
<= 3cm	13 (14.3%)	2 (50.0%)	11 (12.6%)
3.1-5.9 cm	41 (45.1%)	0 (0.0%)	41 (47.1%)
>= 6 cm	37 (40.7%)	2 (50.0%)	35 (40.2%)
Tumor volume	88.23 (293.21)	433.67 (569.60)	76.04 (277.32)
Histology grade			
Poorly diff.	2 (2.3%)	0 (0.0%)	2 (2.4%)
Moderately diff.	68 (77.3%)	3 (75.0%)	65 (77.4%)
Well-diff.	18 (20.5%)	1 (25.0%)	17 (20.2%)
Tumor invasion stage(pT)			
1-2	0 (0.0%)	0 (0.0%)	0 (0.0%)
3	84 (90.3%)	3 (75.0%)	81 (91.0%)
4	9 (9.7%)	1 (25.0%)	8 (9.0%)
Lymph nodes status(pN)			
0	84 (100.0%)	4 (100.0%)	80 (100.0%)
1	0 (0.0%)	0 (0.0%)	0 (0.0%)
2	0 (0.0%)	0 (0.0%)	0 (0.0%)
LN metastasis numbers	0.00 (0.00)	0.00 (0.00)	0.00 (0.00)
Total harvested LNs	22.34 (11.42)	17.67 (15.50)	22.50 (11.34)
LN ratio	0.00 (0.00)	0.00 (0.00)	0.00 (0.00)
Distal margin			

Uninvolved	90 (100.0%)	4 (100.0%)	86 (100.0%)
Involved	0 (0.0%)	0 (0.0%)	0 (0.0%)
Circumferential margin			
Uninvolved	20 (90.9%)	1 (50.0%)	19 (95.0%)
Involved	1 (4.5%)	1 (50.0%)	0 (0.0%)
Cannot be assessed	1 (4.5%)	0 (0.0%)	1 (5.0%)
Lymphovascular invasion			
Absence	84 (90.3%)	4 (100.0%)	80 (89.9%)
Present	9 (9.7%)	0 (0.0%)	9 (10.1%)
Perineural invasion			
Absence	69 (74.2%)	3 (75.0%)	66 (74.2%)
Present	24 (25.8%)	1 (25.0%)	23 (25.8%)
Tumor growth pattern			
Infiltrating	76 (83.5%)	3 (75.0%)	73 (83.9%)
Pushing	15 (16.5%)	1 (25.0%)	14 (16.1%)
Tumor budding			
Absent	39(88.6%)	1 (100.0%)	38 (88.4%)
Present	5 (11.4%)	0 (0.0%)	5 (11.6%)
TRG			
1-2	0 (0.0%)	0 (0.0%)	0 (NA%)
3-5	1 (100.0%)	1 (100.0%)	0 (NA%)
Neoadjuvant CCRT			
No	92 (98.9%)	3 (75.0%)	89 (100.0%)
Yes	1 (1.1%)	1 (25.0%)	0 (0.0%)
dMMR			
Loss	12 (37.5%)	0 (0.0%)	12 (38.7%)
Preserved	20 (62.5%)	1 (100.0%)	19 (61.3%)
BRAF_V600E_Stain			
Negative	14 (82.4%)	0 (NA%)	14 (82.4%)
Positive	3 (17.6%)	0 (NA%)	3 (17.6%)
Alive			
Yes	65 (69.9%)	2 (50.0%)	63 (70.8%)
No	28 (30.1%)	2 (50.0%)	26 (29.2%)
