# Supplementary material for: A Prediction Model for Tumor Recurrence in Stage II–III Colorectal Cancer Patients: From a Machine Learning Model to Genomic Profiling
Source: Biomedicines. 2022 Feb 1;10(2):340. doi: 10.3390/biomedicines10020340 (PMC8961774; doi:10.3390/biomedicines10020340)
Supplement: Supplementary file 1 [file biomedicines-10-00340-s001.zip › Supplementary Table S8. Neoadjuvant Chemoradiotherapy1222V1 .pdf]

Supplementary Table S8. Characteristics of Patients with Neoadjuvant Chemoradiotherapy

		Recurrence		
**Characteristic**	**Overall (N = 120)**	**Yes (n = 26)**	**No (n = 94)**	**P-Value**
yTNM stage				0.499
II	67 (55.8%)	13 (50.0%)	54 (57.4%)	
III	53 (44.2%)	13 (50.0%)	40 (42.6%)	
ypT				0.003
1-2	10 (8.5%)	2 (8.0%)	8 (8.6%)	
3	94 (79.7%)	15 (60.0%)	79 (84.9%)	
4	14 (11.8%)	8 (32.0)	6 (6.5%)	
ypN				0.482
0	53 (55.2%)	11 (45.8%)	42 (58.3%)	
1	30 (31.3%)	10 (41.7%)	20 (27.8%)	
2	13 (13.5%)	3 (12.5%)	10 (13.9%)	
